# Supplementary material for: Flexible Wireless Passive LC Pressure Sensor with Design Methodology and Cost-Effective Preparation
Source: Micromachines (Basel). 2021 Aug 18;12(8):976. doi: 10.3390/mi12080976 (PMC8399622; doi:10.3390/mi12080976)
Supplement: Supplementary file 1 [file micromachines-12-00976-s001.zip › micromachines-1303060-supplementary.pdf]

micromachines

MDPI

*Supplementary Materials*

# Flexible Wireless Passive LC Pressure Sensor with Design Methodology and Cost-effective Preparation

**Zhuqi Sun [1], Haoyu Fang [1], Baochun Xu [1], Lina Yang [1], Haoran Niu [1], Hongfei Wang [1], Da Chen [1,*], Yijian Liu [1,*], Zhuopeng Wang [1], Yanyan Wang [2] and Qiuquan Guo [3]**

1 College of Electronics and Information Engineering, Shandong University of Science and Technology, Qingdao 266590, China; sunzhuqi2021@163.com (Z.S.); fanghy0820@163.com (H.F.); xbcno1@foxmail.com (B.X.); yln_lina@163.com (L.Y.); nhaoran2019@163.com (H.N.); sdkdwhf@163.com (H.N.); sdkdwhf@163.com (H.W.) wzhuopeng@126.com (Z.W.)
2 School of Optoelectronic Science and Engineering Collaborative Innovation Center of Suzhou Nano Science and Technology, Suzhou, 215556, China; yywang@suda.edu.com
3 Shenzhen Institute for Advanced Study, University of Electronics Science and Technology of China, Shenzhen, 518110, China; guoqiuquan@126.com
* Correspondence: chenda@sdust.edu.cn (D.C.); liuyijian@sdust.edu.cn (Y.L.)

**Citation:** Sun, Z.; Fang, H.; Xu, B.; Yang, L.; Niu, H.; Wang, H.; Chen, D.; Liu, Y.; Wang, Z.; Wang, Y.; et al. Flexible Wireless Passive LC Pressure Sensor with Design Methodology and Cost-effective Preparation. *Micromachines* **2021**, *12*, 976. https://doi.org/10.3390/mi12080976

Received: 1 July 2021
Accepted: 11 August 2021
Published: 18 August 2021

**Publisher's Note:** MDPI stays neutral with regard to jurisdictional claims in published maps and institutional affiliations.

**Copyright:** © 2021 by the authors. Licensee MDPI, Basel, Switzerland. This article is an open access article distributed under the terms and conditions of the Creative Commons Attribution (CC BY) license (http://creativecommons.org/licenses/by/4.0/).

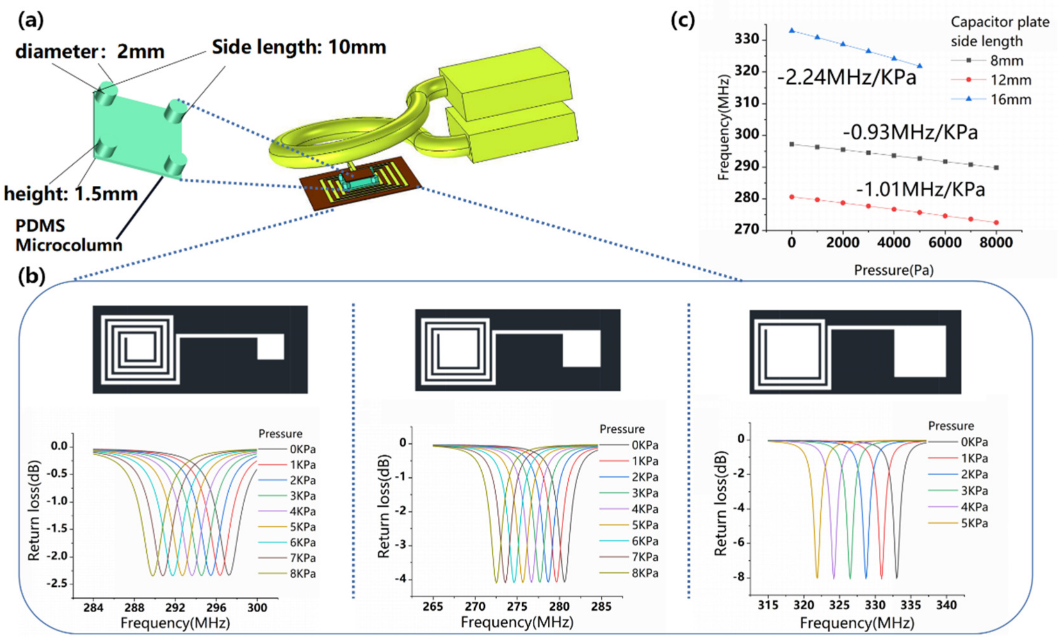


**Figure S1.** Optimization of the dimensional parameters of the LC coil antenna**.** (**a**) Simulation model diagram of the readout system of the LC sensor. (**b**) The resonant frequency curves of the LC pressure sensors with different coil antennas under different pressures. (Line width :1 mm, Line spacing :1 mm) (**c**) Comparison of the pressure sensitivities of the different LC sensors.

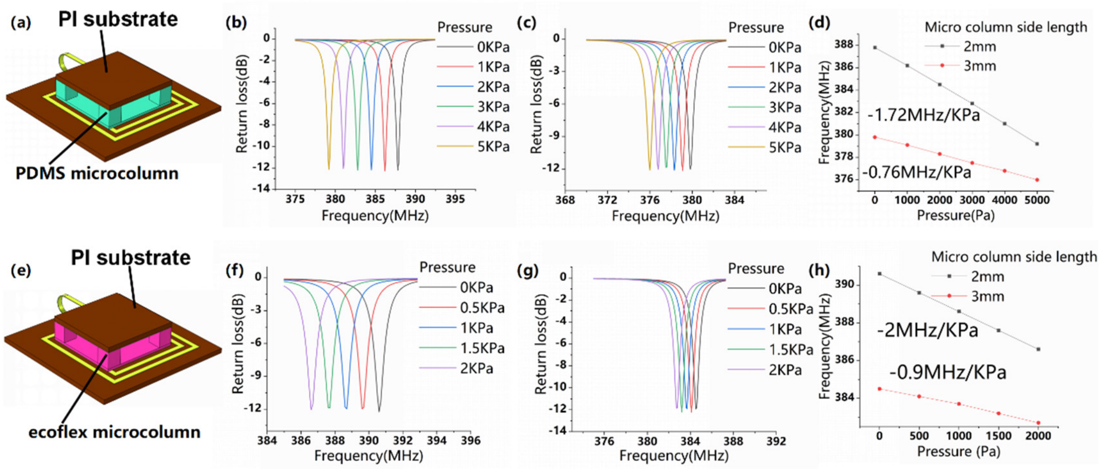


**Figure S2.** Optimization of the dimensional parameters of the capacitor dielectric. (**a**) The LC pressure sensor with PI substrates and PDMS microcolumn dielectric. (**b**) When the side length of the PDMS micro-column is 2 mm, the resonance frequency curves of the sensor under different pressures are depicted. (**c**) When the side length of the PDMS micro-column is 3 mm, the resonance frequency curves of the sensor under different pressures are depicted. (**d**) Comparison of the pressure sensitivities of the LC sensors with PDMS microcolumns with different side lengths. (**e**) The LC pressure sensor with PI substrates and Ecoflex microcolumn dielectric. (**f**) When the side length of the Ecoflex micro-column is 2 mm, the resonance frequency curves of the sensor under different pressures are depicted. (**g**) When the side length of the Ecoflex micro-column is 3 mm, the resonance frequency curves of the sensor under different pressures are depicted. (**h**) Comparison of the pressure sensitivities of the LC sensors with Ecoflex microcolumns with different side lengths.

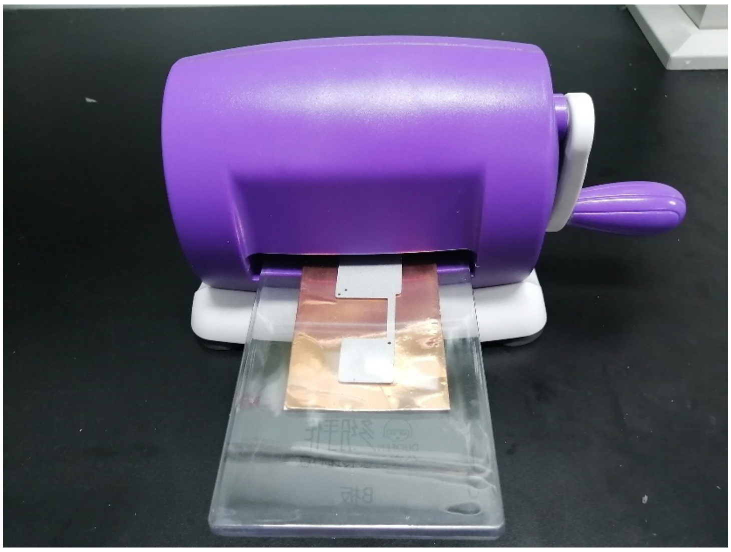

**Figure S3.** The physical image of the embossing process.

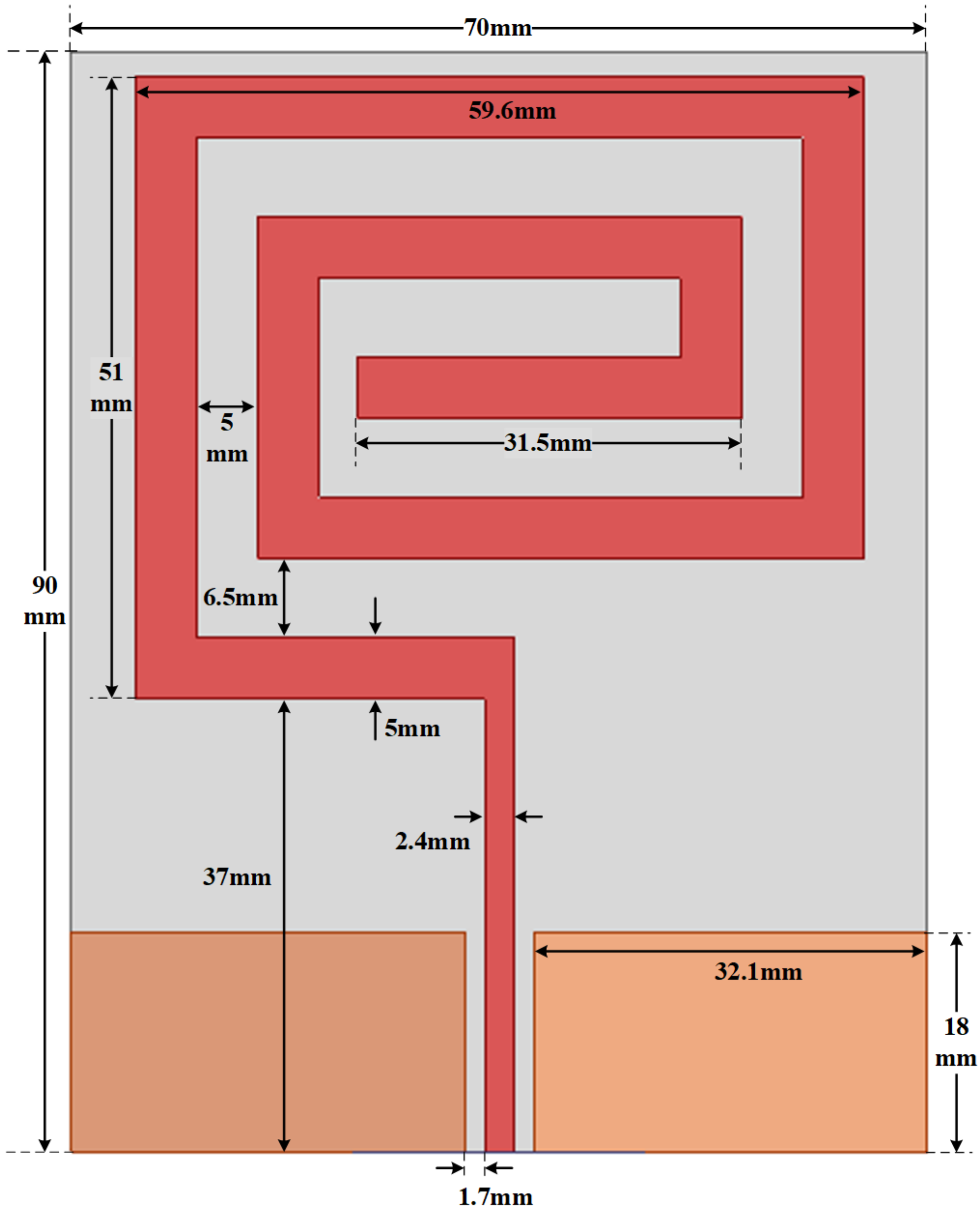


**Figure S4.** Schematic diagram of the monopole antenna with dimension parameters. The planar monopole antenna is made of copper. The dielectric substrate of the planar monopole antenna is made of material FR4 whose thickness is 1.6 mm, and the dielectric constant is 4.4, and the tangent value of loss angle is 0.02.

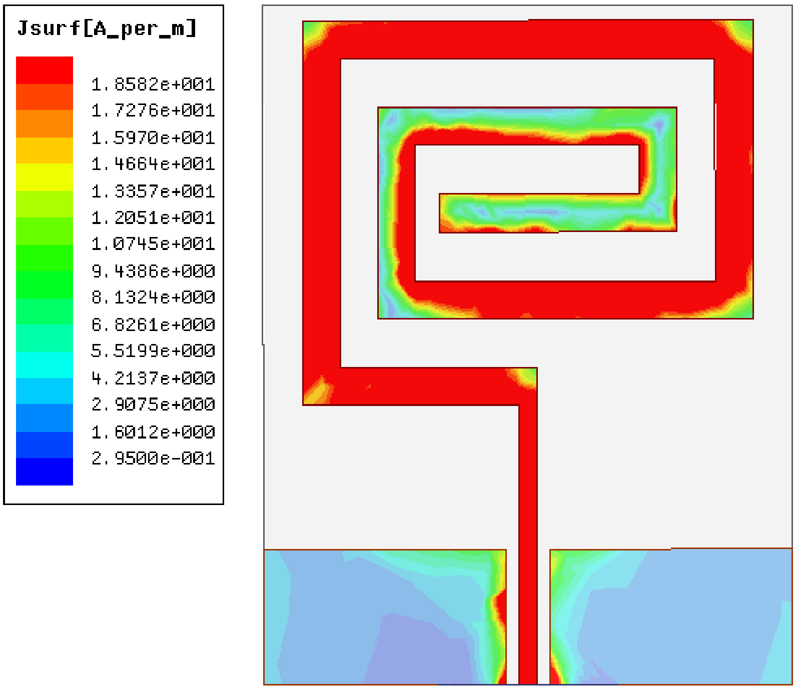


**Figure S5.** Surface current intensity of the planar monopole antenna (HFSS simulation).

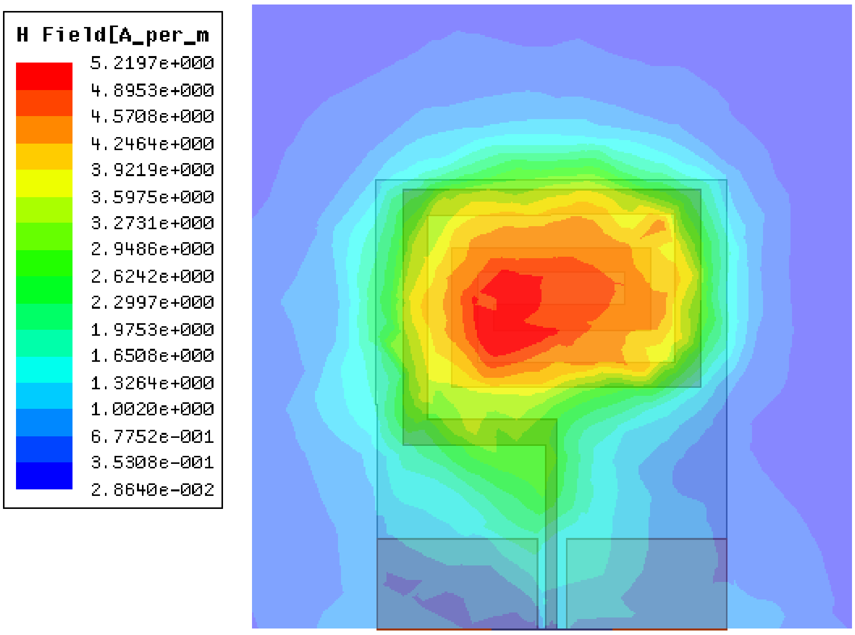


**Figure S6.** The magnetic field strength of the planar monopole antenna at the distance of 10mm above it (HFSS simulation).

**Table S1.** Material parameters of PDMS.

| Parameter | Description | Value |
|---|---|---|
| E | Young's modulus | 750 kPa |
| $V$ | Poisson's ratio | 0.49 |
| $\varepsilon_{\mathrm{r}}$ | Relative permittivity | 2.75 |

**Table S2.** Material parameters of Ecoflex.

| Parameter | Description | Value |
|---|---|---|
| $E_1$ | Young's modulus | 600 kPa |
| $V_1$ | Poisson's ratio | 0.49 |
| $\varepsilon_{\mathrm{r1}}$ | Relative permittivity | 2.3 |
